# Application of neural stem cells in the treatment of intracerebral hemorrhage: a systematic review

**DOI:** 10.3389/fphar.2025.1660614

**Published:** 2025-12-04

**Authors:** Ping Song, Zohaib Shafiq, Hongxiang Jiang, Qiang Cai

**Affiliations:** Department of Neurosurgery, Renmin Hospital of Wuhan University, Wuhan, China

**Keywords:** intracerebral hemorrhage, neural stem cells, transplantation, neural repair, treatment

## Abstract

Stroke ranks among the top global causes of death and disability, with hemorrhagic stroke accounting for 9%–27% of cases. In China, intracerebral hemorrhage (ICH) outpaces Western rates, driving adult mortality and disability. Aging populations and urbanization diversify stroke risk factors, yielding a 28-day ICH mortality rate up to 47% (Greenberg et al., 2022)—sharply higher than ischemic stroke’s 3%—with ∼75% of survivors facing permanent neurological deficits. ICH imposes heavy burdens on individuals, families, and society. Current medical and surgical treatments struggle to enhance long-term outcomes, prompting exploration of neural stem cell (NSC) transplantation. This approach replaces lost neurons via differentiation while offering anti-inflammatory, anti-apoptotic, and neuroprotective benefits. This systematic review evaluates NSC sources, delivery routes, ICH models, therapeutic mechanisms, and early clinical trials, outlining recent progress and future directions for treating hemorrhagic stroke with NSC transplantation.

## 1 Methods

This systematic review was conducted in accordance with the Preferred Reporting Items for Systematic Reviews and Meta-Analyses (PRISMA) guidelines. We searched PubMed, Embase, Web of Science, ClinicalTrials.gov, and the Chinese Clinical Trial Registry (ChiCTR.org.cn) from January 1990 to August 2025 using keywords such as “neural stem cells,” “NSC,” “stem cell transplantation,” “intracerebral hemorrhage,” “ICH,” “hemorrhagic stroke,” and combinations thereof. Inclusion criteria were: English-language studies (preclinical or clinical) on NSC application in ICH, published peer-reviewed articles, and full-text availability. Exclusion criteria included case reports, reviews, non-ICH stroke studies, and non-NSC stem cell types. Two independent reviewers screened titles/abstracts and full texts, resolving disagreements by consensus. Data extraction included study design, NSC source/route/dose, outcomes, and mechanisms. Quality assessment used SYRCLE’s risk of bias tool for preclinical studies and Cochrane for clinical trials. A PRISMA flow diagram (not shown here; to be included in final submission) identified 1,250 records, with 68 studies included after duplicates and screening. No meta-analysis was performed due to heterogeneity.

## 2 Introduction

ICH is spontaneous bleeding within the brain, often triggered by hypertension, cerebral amyloid angiopathy, or vascular anomalies such as aneurysms, arteriovenous malformations, Moyamoya disease, or vasculitis (Kim et al., 2022). This devastating condition carries an early mortality rate of 30%–47% (Greenberg et al., 2022) and leaves about two-thirds of patients either dead or permanently disabled (Chen et al., 2020). Current ICH treatments aim to prevent re-bleeding and reduce secondary brain injury, but the nervous system’s limited regenerative capacity results in persistently poor prognoses. Repairing damaged neural tissue or stimulating neurogenesis offers a promising strategy to improve outcomes for ICH patients.

Standard treatments for primary and secondary brain injuries following ICH include surgery, intracranial pressure management, symptomatic care, and rehabilitation. However, these approaches have shown only modest success, highlighting the need for innovative therapies. Stem cell therapy has emerged as a compelling option, gaining attention from researchers (Tuazon et al., 2019; Gao et al., 2018). Stem cells are unique in their ability to proliferate, self-renew, and differentiate into various cell types under specific conditions. They are broadly classified into embryonic stem cells (ESCs) and adult stem cells (ASCs)—also termed somatic stem cells (SSCs)—found in tissues like the brain (neural stem cells, NSCs), blood (hematopoietic stem cells, HSCs), and bone marrow (mesenchymal stem cells, MSCs). NSCs, located in the nervous system, can divide symmetrically or asymmetrically to produce central nervous system (CNS) cell types, including neurons, astrocytes, and oligodendrocytes. First identified by Reynolds and Weiss in 1992 through isolation from adult mouse striatum (Reynolds et al., 1992), NSCs exhibit proliferative and multipotent potential. They can remain quiescent for extended periods, activating in response to injury to support tissue regeneration and cell replacement (Gao et al., 2018). Recent work by Petrelli et al. (2023) underscores the role of mitochondrial metabolism—specifically the mitochondrial pyruvate carrier (MPC)—in regulating adult neural stem/progenitor cell (NSPC) activity, revealing how metabolic cues influence whether NSPCsremain dormant or activate for neuroregeneration.

NSC transplantation holds significant therapeutic potential due to its ability to suppress inflammation, differentiate into neurons, and promote neuroprotection and neurogenesis key features that make it a promising treatment for ICH. This review synthesizes current research on NSCs in ICH therapy, focusing on their sources, administration routes, and clinical implications.

## 3 Sources of neural stem cells

NSCs used for injury repair can be classified into endogenous and exogenous sources. Current research indicates that both activating dormant endogenous NSCs and transplanting exogenous NSCs can secrete neurotrophic factors to improve their growth microenvironment, suppress inflammation, enhance recovery, and differentiate into neurons to restore lost neurological function in damaged areas.

### 3.1 Endogenous source of neural stem cells

The traditional view that NSCs exist only during embryonic development and lack regenerative capacity after neural injury has been overturned by accumulating evidence. In 1992, Reynolds and Weiss isolated self-renewing, multipotent NSCs from the striatum of adult mice (Reynolds et al., 1992), sparking further discoveries of NSCs in mammals, birds, and humans, where they exhibit directed migration, proliferation, and differentiation (Lendahl, 2022). These cells predominantly reside in the hippocampal dentate gyrus and the brain’s subventricular zone key regions for NSC research (Gao et al., 2018; Martinez-Galdamez et al., 2019; Mitrecic et al., 2022). Typically quiescent in healthy states, endogenous NSCs activate and migrate to injury sites under pathological conditions. For instance, Gao et al. stimulated endogenous NSCs in ICH rat models to support neuronal repair, with signs of neurogenesis observed in tissues near hematomas in human ICH patients (Gao et al., 2018). Similarly, repeated transcranial magnetic stimulation has been shown to boost NSC proliferation and differentiation via the MAPK signaling pathway post-ICH (Cui et al., 2019). These responsive NSCs, termed endogenous stem cells, offer a safe therapeutic avenue due to their lack of immune rejection and tumorigenic risk (Rodriguez-Frutos et al., 2016). Yu et al. found that upregulating the hypoxia-inducible factor-1α (HIF-1α) gene enhances NSC proliferation, migration, and differentiation, aiding neural recovery in ICH patients (Yu et al., 2013).Physical exercise has even been shown to enhance NSC survival and migration following ICH.These findings highlight NSCs’ neuroplasticity and regenerative potential, supported by their low immunogenicity and compatibility with brain tissue. However, the specific factors triggering NSC differentiation remain unclear (Zhao et al., 2022; Zhou et al., 2022), and their limited numbers and migration challenges restrict their use as a primary ICH treatment strategy.

### 3.2 Exogenous sources of neural stem cells

Exogenous NSCs are derived from primary tissue cultures, pluripotent stem cell differentiation, or somatic cell reprogramming (Tuazon et al., 2019; Gao et al., 2018). Advances in microdissection have simplified NSC isolation from embryonic tissues, with Krutika et al. successfully extracting NSCs from whole mouse brains (Deshpande et al., 2019) and Zhao Quanjun et al. refining primary culture techniques to obtain NSCs from discarded brain tissue of patients with acute traumatic brain injury, later inducing neuronal differentiation (Quanjun et al., 2019). While feasible, primary tissue culture faces challenges such as low cell yield, limited differentiation efficiency, and the need to maintain stable NSC passages without mutations or senescence. Emerging techniques such as growth factor use, 3D cultures, and microRNA-induced differentiation are improving scalability and safety for clinical applications (Tang et al., 2017). Pluripotent stem cells, though unable to form complete organisms, can differentiate into NSCs and other cell types (Wu et al., 2017). These cells are typically sourced from embryonic tissues or somatic cell nuclear transfer, but ethical concerns, immune rejection risks, and potential tumorigenicity make embryonic sources less ideal (Zhao et al., 2022). Induced pluripotent stem cells (iPSCs), pioneered by Yamanaka through somatic cell reprogramming (Takahashi and Yamanaka, 2006), mimic embryonic stem cells’ pluripotency and serve as a key NSC source. However, producing high-purity NSCs from iPSCs is time-intensive and poses safety risks (Yang et al., 2017).

Viral vectors, the primary method for delivering transgenes, raise concerns about tumorigenesis, complicating clinical translation (Mollinari and Merlo, 2021). Transdifferentiation, the direct conversion of one differentiated cell type into another via selective gene expression (Erharter et al., 2019), offers an alternative approach. Vierbuchen et al. transformed mouse fibroblasts into neurons using transcription factors Ascl1, Brn2, and Myt1l (Vierbuchen et al., 2010), while Hermann et al. employed a two-step process to convert mesenchymal stem cells (MSCs) into NSCs and then neurons (Hermann et al., 2006). Direct transdifferentiation to neurons is simpler but also yields NSCs as a byproduct.

## 4 Neural stem cell transplantation routes

**FIGURE 1:**
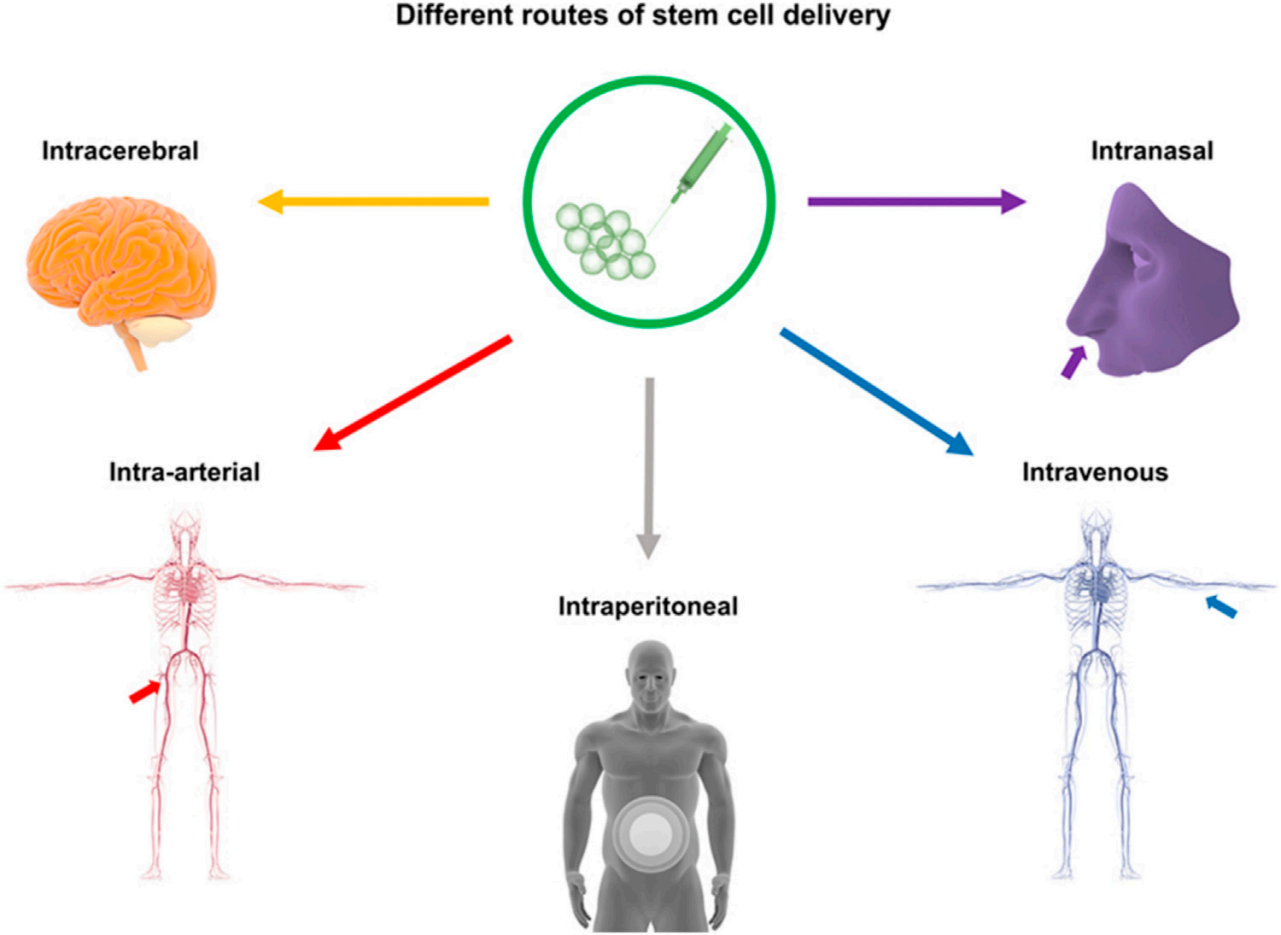
Routes of Stem Cell Transplantation: This figure illustrates stem cell delivery pathways for stroke treatment, including intracerebral (stereotactic injection), intravascular (venous/arterial), intraperitoneal, intraventricular, intravenous, intranasal, and intrathecal methods.

Currently, there are several pathways for NSC transplantation, including intracerebral transplantation, intrathecal administration, intravascular injection, and intranasal administration. The most commonly used method is intracerebral transplantation, through intracerebral transplantation, intravascular injection, and intracerebral transplantation via the cerebrospinal fluid (CSF) route (Tuazon et al., 2019; Zhao et al., 2022). However, each method has its own advantages and disadvantages, with no optimal transplantation route identified at present (Figure 1). (See Table 1 for a summary of key translational parameters, including routes, time windows, and doses from preclinical and clinical studies.).

**TABLE 1:** Summary of key translational parameters in NSC studies for ICH.

Parameter	Preclinical examples	Clinical examples	Notes/Limitations
Delivery Routes	Stereotactic (e.g., hematoma cavity (Gao et al., 2018)); Intravascular (venous (Vahidy et al., 2016)); CSF (ventricular (Cruz-Martinez et al., 2017)); Intranasal (Sun et al., 2015)	Intravenous (e.g., MSCs in (Ahn et al., 2018)); Intraventricular (Chang et al., 2016)	Preclinical favors targeted routes; clinical prioritizes minimally invasive; heterogeneity leads to variable efficacy
Time Windows	Acute (1–3 days post-ICH (Zhang et al., 2006)); Subacute (1 week (Cui et al., 2019))	Subacute/chronic (weeks to >1 year (Tsang et al., 2017))	Early timing reduces secondary injury but risks inflammation; optimal window unclear due to model differences
Cell Doses	10^5–10^7 cells (e.g., 5 × 10^5 in rats (Cui et al., 2019))	10^7–10^8 cells (e.g., 2 × 10^7 MSCs (Ahn et al., 2018))	Balances efficacy vs. risks (thrombosis, tumorigenesis); dose-response studies lacking
Outcomes	Improved neurogenesis, reduced deficits (Gao et al., 2018; Zhou et al., 2022)	Safety/feasibility; modest functional gains (Ahn et al., 2018; Chang et al., 2016; Tsang et al., 2017)	Preclinical overestimates due to healthy models; clinical limited by small samples

### 4.1 Stereotactic injection

Stereotactic injection, or intracerebral transplantation, delivers NSC suspensions directly into the hematoma cavity, offering the most targeted approach among transplantation methods. However, blood degradation products and reactive astrocytes in the cavity can trigger NSC death. While NSCs differentiate effectively *in vitro*, *in vivo* outcomes are less promising, possibly due to high local cell density impeding differentiation. To improve survival, researchers have developed genetically modified, traceable, anti-apoptotic NSCs (Patent: CN202111510957.7, March 1, 2022). Others suggest co-administering neurotrophic factors during injection to enhance NSC viability, though these strategies await further validation (Gao et al., 2018; Zhou et al., 2022).

### 4.2 Intracerebrospinal fluid route transplantation

Transplantation via the CSF route includes lumbar puncture, ventricular puncture, and cisterna magna injection. Lumbar puncture delivers NSCs into the subarachnoid space, enabling circulation throughout the brain via CSF. Ventricular puncture injects NSC suspensions into the lateral ventricle, allowing rapid distribution across ventricles and brain tissue while potentially stimulating endogenous NSC proliferation through secreted factors (Cruz-Martinez et al., 2017). Cisterna magna injection, limited to animal models due to its risk of brainstem damage, has not been reported in clinical use.

### 4.3 Intravascular injection

Intravascular injection of NSCs can be venous or arterial. Venous delivery sends NSCs through systemic circulation, leading to substantial cell loss, with many trapped in the liver and lungs (Vahidy et al., 2016), though multiple injections could theoretically boost survival rates. Arterial injection (typically via the carotid artery) provides direct brain access but poses technical challenges and risks thrombosis due to NSC aggregation (Kim et al., 2022; Gao et al., 2018). Both intravascular and CSF routes face challenges: poor NSC migration, the blood-brain barrier (BBB), and lack of targeted guidance limit cell accumulation in the hematoma cavity. Solutions like targeting molecules or nanomaterial carriers could enhance delivery precision. As NSC transplantation strategies diversify, their impact on neural repair varies, and while experimental progress continues, identifying the safest, most effective routes remains a research priority. Advances in genetics, neurophysiology, and novel therapeutics promise standardized clinical applications soon (Tuazon et al., 2019; Zhao et al., 2022).

Alternative NSC transplantation routes include intranasal administration, which minimizes secondary damage. Sun et al. (Sun et al., 2015) detected hypoxia-preconditioned mesenchymal stem cells (MSCs) in ICH brain tissue following intranasal delivery. Other options, such as abdominal delivery and nanomaterial-enhanced combined routes, aim to improve transplantation efficiency (Tuazon et al., 2019; Gao et al., 2018) (Figure 1).

## 5 ICH animal model studies

Preclinical research aims to enhance clinical efficacy, prognosis, and quality of life, focusing heavily on constructing animal models to simulate ICH and evaluate SC interventions. These models strive to replicate key human ICH pathophysiological processes, such as hematoma expansion and recurrence. While insights into animal ICH mechanisms grow, future efforts will likely prioritize translating findings to human studies. Given ICH’s complex primary and secondary injury mechanisms, experimental models are vital for elucidating pathophysiology and developing treatments. Established models include autologous blood injection, collagenase injection, intracerebral balloon inflation, and cerebrovascular injury, alongside less common approaches. Rodents (rats and mice) and pigs dominate studies, with rats used in 63% of cases and the autologous blood injection model leading at 51% (Paiva et al., 2023). These models enhance understanding of intracranial pressure, neuroinflammation, immunity, and cerebral hemodynamics, informing treatment strategies and boosting clinical diagnostic and therapeutic capabilities. Table 2 summarizes the main models’ advantages and disadvantages.

**TABLE 2:** Comparison of major ICH models.

Model method	Advantages	Disadvantages
Autologous blood injection	High repeatability, uniform hematoma size (e.g., 50–100 μL in rats (Liu et al., 2015)), clinically relevant	No vessel rupture simulation, re-bleeding hard to assess, varies by blood source
Bacterial collagenase	Enables hematoma expansion and edema studies; mimics rupture	Diffuse bleeding, excessive inflammation, unfit for acute ICH
Brain vascular injury	Replicates vascular rupture and some aspects of blood toxicity	Risks ischemia, craniotomy alters ICP
Intracerebral balloon	Precise space-occupying effect (e.g., 0.05 mL inflation (Sinar et al., 1987))	Lacks blood toxicity simulation
Spontaneous ICH	Mirrors clinical ICH (80% incidence in hypertensive rats (Ogata et al., 1980))	Slow to develop, costly, hard to detect

This table compares ICH, model construction methods by their strengths and limitations.

### 5.1 Autologous blood intracerebral injection model

Since the 1960s, the autologous blood intracerebral injection model has offered a straightforward, effective method to create brain parenchymal hematomas by injecting blood into the frontal lobe or basal ganglia. Initially developed in larger animals (e.g., cats, dogs, pigs, sheep, monkeys) (Cao et al., 2016), it enables assessment of hemolysis-induced toxicity, immune-inflammatory responses, and intervention efficacy. Its primary advantage is producing uniform hematoma sizes, mimicking rapid blood accumulation in acute clinical ICH (Liu et al., 2015). However, it fails to replicate spontaneous vascular rupture and often results in blood reflux along the needle track (Yang et al., 1994). By adjusting injected volume, it simulates intracranial hypertension’s biochemical and pathophysiological effects, creating hematomas with varying mass effects (Manaenko et al., 2011). Typically a single injection, the model was refined in 1996 by Deinsberger’s “two-injection method” (Deinsberger et al., 1996): a small initial injection (e.g., into the basal ganglia) forms a clot, followed by a second injection to prevent backflow, a technique widely adopted in small animal studies. Limitations include its inability to model spontaneous bleeding, vascular rupture, or rebleeding (Gao et al., 2018; Yang et al., 1994).

### 5.2 ICH collagenase model

Bacterial collagenase, a metalloproteinase, degrades collagen in the extracellular matrix and basement membranes around brain capillaries, weakening vessels and inducing hemorrhage (Manaenko et al., 2011). Introduced in mice in the early 1990s (Rosenberg et al., 1990), this model excels in studying hematoma expansion, vasogenic edema, anticoagulation effects, axonal degeneration, iron-mediated apoptosis, endothelial dysfunction, and BBB damage. Compared to the autologous blood injection model, it produces more pronounced pathological effects despite sharing similar injury mechanisms (Paiva et al., 2023). Bleeding is diffuse with a gradual hematoma onset, differing from acute clinical hematomas. Typically, researchers inject bacterial type IV collagenase though types VI, VII S, or XI are also used over 2–16 min (Paiva et al., 2023). Preferred for small animals like rats and mice, it effectively mimics hematoma expansion and edema, aiding investigations into ICH-related brain edema, neurological recovery, drug efficacy, and homeostasis-modulating therapies (Jing et al., 2019). It also simulates vascular rupture, supporting long-term outcome studies. However, its early and prolonged inflammatory response may exaggerate inflammation, complicating mechanistic studies, while excessive bleeding risks unintended ischemic damage, potentially worsening neurological deficits and recovery (Zhou et al., 2013).

### 5.3 ICH cerebrovascular injury model

The cerebrovascular injury model induces cortical bleeding in rats by exposing cortical veins via craniotomy and puncturing them with a bent needle (Xue and Del Bigio, 2003). Compared to blood infusion and collagenase models, it shows distinct inflammatory, molecular, and cellular responses (Xue and Del Bigio, 2003). Peak DNA damage in neurons and CD8-reactive lymphocytes occurs at 3 days post-injury, with sustained microglia/macrophage activation from 3 to 7 days, though only minor neuronal death persists by 21–28 days. Neutrophil counts are notably lower than in the other models (Xue and Del Bigio, 2003). Alternatives include laser-induced microbleeds to study coagulation (Lauer et al., 2011) and ultrasound-guided middle cerebral artery puncture in dogs, achieving high success (Zhou et al., 2013). Limited to open craniotomy, this model tends to cause less severe brain damage.

### 5.4 ICH intracerebral balloon model

This rat ICH model uses a microsphere catheter method (Sinar et al., 1987). A 25 Fr microballoon, mounted on a needle, is inserted into the right caudate nucleus via a skull burr hole, inflated to 0.05 mL in 20 s, and held for 10 min before deflation. Post-study analyses of brain histology, intracranial pressure (ICP), and cerebral blood flow reveal reduced flow and elevated ICP, confirming effective injury. The model’s key strength is mimicking the space-occupying effect of acute human ICH (Alharbi et al., 2016), making it ideal for studying mechanical brain injury. However, limitations include: (1) no replication of blood toxicity, reducing ischemia compared to blood injection models; (2) no blood-brain barrier disruption; and (3) no edema formation (Paiva et al., 2023).

### 5.5 Spontaneous ICH model

Hypertension, a leading cause of human cerebrovascular disease, underpins this model, where genetically manipulated hypertensive rats develop spontaneous vascular rupture resembling clinical ICH. Inbreeding achieves an 80% ICH incidence (Ogata et al., 1980). Its strength is replicating human spontaneous ICH and hypertension-related stroke pathophysiology. Drawbacks include lengthy breeding times, high costs, and risks of inbreeding-related genetic variability, restricting its use.

### 5.6 Other ICH models

#### 5.6.1 High salt method

High sodium intake elevates blood pressure, disrupting cerebral vessel tight junctions and causing small, scattered hematomas similar to clinical cases (Appel, 2014). IIscts drawback is the prolonged time to results, delaying research.

#### 5.6.2 Ischemia induction method

Occluding the middle cerebral artery induces ischemia, followed by CO2 and anticoagulants to trigger hemorrhage in the ischemic zone, ideal for studying post-stroke rebleeding.

#### 5.6.3 Trauma-induced ICH method

Violent trauma creates intracerebral hematomas but is hard to control, with high variability, poor reproducibility, and frequent animal mortality, limiting it to traumatic ICH simulation.

#### 5.6.4 Composite and other methods

Combining methods (e.g., with heparin) enhances ICH models, while ultrasound or angiography-guided vessel punctures offer additional approaches (Zhou et al., 2013).

### 5.7 Limitations of animal models and translational relevance

Although healthy animal models provide valuable insights, they do not fully replicate clinical ICH pathophysiology. For example, the collagenase model exaggerates acute inflammation (e.g., higher IL-1β/TNF-α levels than in humans) and diffuse bleeding, while autologous blood models fail to capture spontaneous vascular rupture or rebleeding, leading to overestimation of NSC efficacy in controlled environments (Paiva et al., 2023; Zhou et al., 2013). Human ICH often involves comorbidities like hypertension, diabetes, or aging, absent in young rodent models, which may reduce generalizability—e.g., comorbidities could impair NSC migration or survival. These discrepancies highlight the need for advanced models (e.g., aged/comorbid animals) to bridge the translational gap and better predict clinical outcomes.

## 6 Impact of ICH pathophysiology on NSCs

ICH pathophysiology arises from hematoma compression and ongoing damage by blood and its breakdown products, categorized as primary brain injury (PBI) and secondary brain injury (SBI). In experimental animals, prognosis hinges on: (1) the hematoma’s space-occupying effect and resulting ischemia-hypoxia; (2) thrombin toxicity and plasminogen activation with fibrinolysis; (3) SBI from red blood cell lysis and metabolic byproducts; and (4) tissue damage from clots and inflammatory/complement cascades. These factors highlight key considerations for NSC transplantation:

### 6.1 Cell quantity

The optimal NSC dose for ICH remains unclear, varying by cell type and delivery route. Most studies use millions of cells, balancing efficacy against risks like thrombosis or tumorigenesis from excessive doses (Gao et al., 2018).

### 6.2 Timing

Early transplantation is favored (Auriat et al., 2011), reducing SBI (e.g., inflammation, apoptosis) within the first week post-ICH to aid recovery. Zhang et al. found MSC transplantation on day 3 outperformed days 1, 5, or 7, linking early hematoma clearance to better cell survival (Zhang et al., 2006).

### 6.3 Immunomodulation

While some studies report no acute rejection in autologous settings, evidence of immune rejection exists in allogeneic NSC transplantation (e.g., T-cell mediated responses), necessitating further research on immunosuppressive therapy (Pluchino et al., 2005).

## 7 NSCs neurorepair mechanisms

### 7.1 Replacement effect

Experiments demonstrate that NSC transplantation improves neurological deficits by differentiating into neurons and glial cells to replace lost neurons. These new neurons form functional synaptic connections with existing ones a process termed the replacement effect (Ceto et al., 2020). However, subsequent studies suggest this mechanism alone cannot fully account for functional improvement, as NSC-derived neurons exhibit limited synaptic integration, and their signal transmission efficacy remains unconfirmed (Wen et al., 2014).

### 7.2 Bystander effect

**FIGURE 2:**
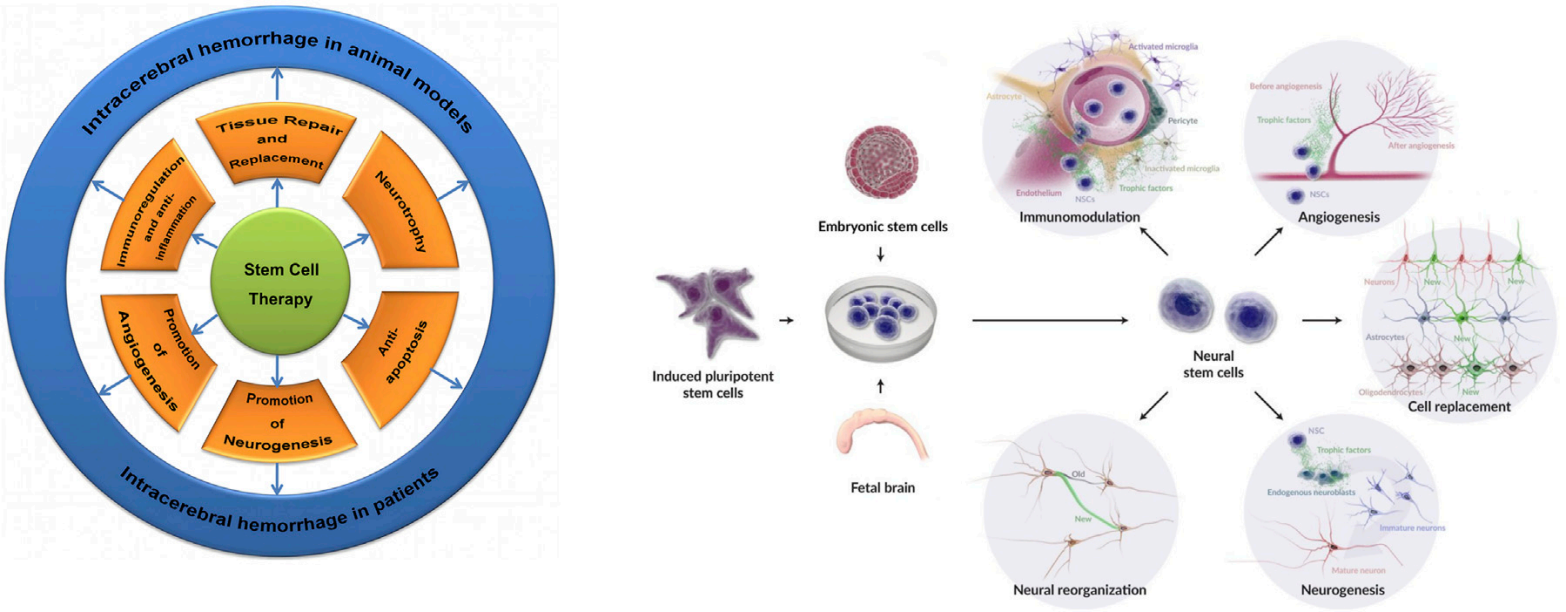
Stem cell and NSC therapeutic mechanisms in ICH: Illustrates protective roles including repair, replacement, neurotrophic support, anti-apoptosis, anti-inflammation, vascular regeneration, neurogenesis, immunomodulation, neural repair, differentiation into astrocytes/neurons/oligodendrocytes, and host tissue integration—culminating in enhanced tissue repair and neurological recovery.

NSCs reduce host cell death by secreting neurotrophic factors, boosting endogenous repair, promoting vascular regeneration, and suppressing inflammation a mechanism known as the bystander effect (Reynolds et al., 1992; Huang and Zhang, 2019). Specific pathways include downregulation of pro-inflammatory cytokines (e.g., IL-1β, IL-6, TNF-α) via NF-κB inhibition. NSC-derived exosomes, small vesicles containing RNA and proteins, may drive this effect, enhancing autophagy, reducing neuronal apoptosis, and curbing inflammation with their neurotrophic cargo (Rong et al., 2019). In a mouse ICH model, gene-modified NSCs overexpressing BDNF, GDNF, Akt1, and VEGF enhanced neurological recovery, likely by improving vascular regeneration, repairing the blood-brain barrier (BBB), and reducing edema (Gao et al., 2018) (Figure 2), though the precise mechanisms await further exploration (Pasi et al., 2021).

The treatment of ICH shows limited effectiveness in reducing damage and improving the recovery of neurological function, often resulting in persistent severe neurological deficits. Given the complex molecular mechanisms underlying this pathology, exploring appropriate and effective treatment methods is of significant importance (Li et al., 2022). In animal models and patients, the therapeutic mechanisms of Stem Cells (SCs) in ICH involve tissue repair and replacement, neural nutrition, anti-apoptotic effects, anti-inflammatory properties, promotion of vascular regeneration, and neurogenesis (Gao et al., 2018). Transplantation of NSCs derived from ESCs, iPSCs, or fetal brain into veins, parenchyma, or brain ventricles has shown various therapeutic functions (Baker et al., 2019).

## 8 Basic research on NSCs therapy for hemorrhagic stroke

### 8.1 Immunomodulation and inflammatory response regulation by NSCs

Early studies linked post-ICH recovery from neural NSC transplantation to neuron replacement and network formation. Recent data emphasize inflammation’s pivotal role in stroke-induced brain damage, tying neuronal injury severity to immune activation (Lee et al., 2007). NSC-driven recovery may hinge more on immune modulation than cell replacement. In experimental autoimmune encephalomyelitis models, transplanted NSCs bolstered neuroprotection by curbing T-cell activity and triggering T-cell apoptosis (Pluchino et al., 2005). Post-stroke, activated microglia—brain-resident immune cells—release pro-inflammatory cytokines (e.g., IL-1β, IL-6, TNF-α) within minutes, amplifying damage by breaching the blood-brain barrier (BBB). NSCs likely dampen this cascade through a bystander effect, with studies affirming their anti-inflammatory, neuroprotective effects (Baker et al., 2019). This involves specific mediators like suppression of NF-κB and JAK-STAT pathways.

Genetically modified human NSCs (hNSCs) overexpressing BDNF, GDNF, Akt1, and VEGF markedly enhance neurological recovery in ICH mice (Zhou et al., 2022). Boosting post-stroke endogenous neurogenesis also shows promise. In ICH rats, repeated transcranial magnetic stimulation directed NSC differentiation toward neurons, minimizing glial-like outcomes and aiding functional recovery (Cui et al., 2019). NSC transplantation amplifies this by spurring endogenous NSC proliferation in the subventricular zone (SVZ) and dentate gyrus (DG), driving neural progenitor migration to damaged areas for neuronal differentiation. Though exact mechanisms remain elusive, neurotrophic and regenerative growth factors likely underpin tissue repair and immune-inflammatory suppression (Eriksson et al., 1998).

### 8.2 NSCs’ role in replacement and neurotrophic functions

NSCs differentiate into mature neurons and integrate into host brain tissue, functioning as cell replacement therapy while also delivering neurotrophic support. Transplanted NSCs express the neuroblast marker Doublecortin (DCX) within 2 months, with levels declining as they become neurons or glial cells expressing markers like NeuN, HuD, MAP2, and βIII-tubulin (Tornero et al., 2013). Their replacement role’s timing and scope remain uncertain (Baker et al., 2019). Animal studies show NSCs reduce secondary damage via anti-inflammatory effects, enhance endogenous neurogenesis and synaptic remodeling, and serve as cell substitutes (Baker et al., 2019). However, rapid spontaneous recovery in rodent models complicates distinguishing neurotrophic benefits from integration effects 2–3 months post-transplantation (Tornero et al., 2013). Whether NSC differentiation is driven by intrinsic cues, host environment, or occurs spontaneously requires further investigation.

### 8.3 NSCs’ promotion of angiogenesis

NSCs promote angiogenesis, driving neural repair through synaptic reorganization and neurogenesis. Post-stroke, nutrient and growth factor exchange between endothelial cells and migrating endogenous NSCs supports vascular regeneration. Rodent studies show transplanted NSCs boost microvessel density and angiogenic receptor expression in the ischemic penumbra, enhancing angiogenesis (Baker et al., 2019). This process hinges on VEGF signaling produced by NSCs or amplified by host tissue closely tied to neurological recovery (Ryu et al., 2016). Embryonic NSCs also express Ang1, increasing microvessel counts post-transplantation (Hicks et al., 2013). Effective vascular networks in the penumbra depend on neurovascular units (NVUs), which NSC therapy strengthens by upregulating tight junction proteins (claudin, occludin, ZO1) and dystroglycan, while reducing BBB leakage (Horie et al., 2011). However, while NSC therapy enhances angiogenesis via VEGF signaling, it may transiently exacerbate BBB leakage in the acute phase, potentially worsening edema. Studies suggest that balanced upregulation of tight junction proteins mitigates this risk (Horie et al., 2011), but further research is needed to optimize timing and dosing to prevent adverse effects.

## 9 Clinical trials

SC transplantation holds significant promise for stroke treatment, yet research predominantly targets ischemic stroke, with ICH receiving less attention and limited clinical trial validation. SC therapies for neuroregeneration are classified into three generations:

### 9.1 First generation

Utilizes adult stem cells hematopoietic stem cells (HSCs), mesenchymal stem cells (MSCs), and fetal-derived NSCs offering immunomodulation, anti-inflammatory, angiogenic, anti-apoptotic, and trophic benefits. These are primarily studied for autoimmune diseases, neurodegeneration, heart failure, skeletal disorders, and gastrointestinal conditions (Ratcliffe et al., 2013).

### 9.2 Second generation

Employs embryonic stem cells (ESCs) and induced pluripotent stem cells (iPSCs) for their unlimited self-renewal and pluripotency, with trials focusing on spinal cord injuries, retinal degeneration, type 1 diabetes, and ischemic heart failure (Kim et al., 2022).

### 9.3 Third generation

Enhances efficacy by engineering first- and second-generation SCs as drug carriers to optimize outcomes (Kim et al., 2022).

Only six SC transplantation trials for hemorrhagic stroke are registered on ClinicalTrials.gov: two completed, two ongoing, and two with unreported results. A South Korean phase I trial demonstrated that MSC transplantation for severe intraventricular hemorrhage in preterm infants reduced periventricular damage in some cases, with no deaths or severe adverse effects, suggesting safety and feasibility (Ahn et al., 2018). Chang et al. found that bone marrow or umbilical cord MSC transplantation in patients with moderate-to-severe ICH deficits (over 60 months) outperformed surgical hematoma evacuation alone in functional outcomes (Chang et al., 2016). Tsang et al.’s phase I/II randomized controlled trials showed autologous bone marrow MSC transplantation improved neurological recovery compared to placebo (Tsang et al., 2017). Conversely, the Chinese Clinical Trial Registry (www.chictr.org.cn) lists 16 NSC trials, mostly for ischemic stroke, including exosome therapy, cerebral palsy, stroke sequelae, and spinal cord injuries, conducted as single- or multi-center studies. SCs, often sourced from accessible umbilical cord or bone marrow, are typically administered late (weeks to over a year post ICH) rather than acutely, reflecting the priority of life-saving interventions in the initial phase. (See Table 3 for a summary of key clinical trials.).

**TABLE 3:** Summary of key clinical trials on stem cell therapy for ICH.

Trial ID	Phase	Cell type	Route	Patients (n)	Primary endpoints	Status/Results
NCT02150564 (ClinicalTrials.gov)	I	MSCs	Intravenous	10	Safety, feasibility	Completed; Safe, reduced damage (Ahn et al., 2018)
ChiCTR-OPC-16008286	I/II	MSCs	Intraventricular	20	Neurological recovery (mRS)	Completed; Improved outcomes vs. surgery (Chang et al., 2016)
NCT01371305	I/II	Bone marrow MSCs	Intravenous	100	Functional improvement (NIHSS)	Completed; Better recovery vs. placebo (Tsang et al., 2017)
NCT04261387	II	iPSC-derived NSCs	Intrathecal	30	Safety, efficacy	Ongoing; Preliminary safety data
ChiCTR2000037944	I	Fetal NSCs	Intravenous	15	Adverse events, neurogenesis	Unreported
NCT04097652	I	MSCs (umbilical cord)	Intranasal	12	BBB integrity, inflammation	Ongoing

**FIGURE 3:**
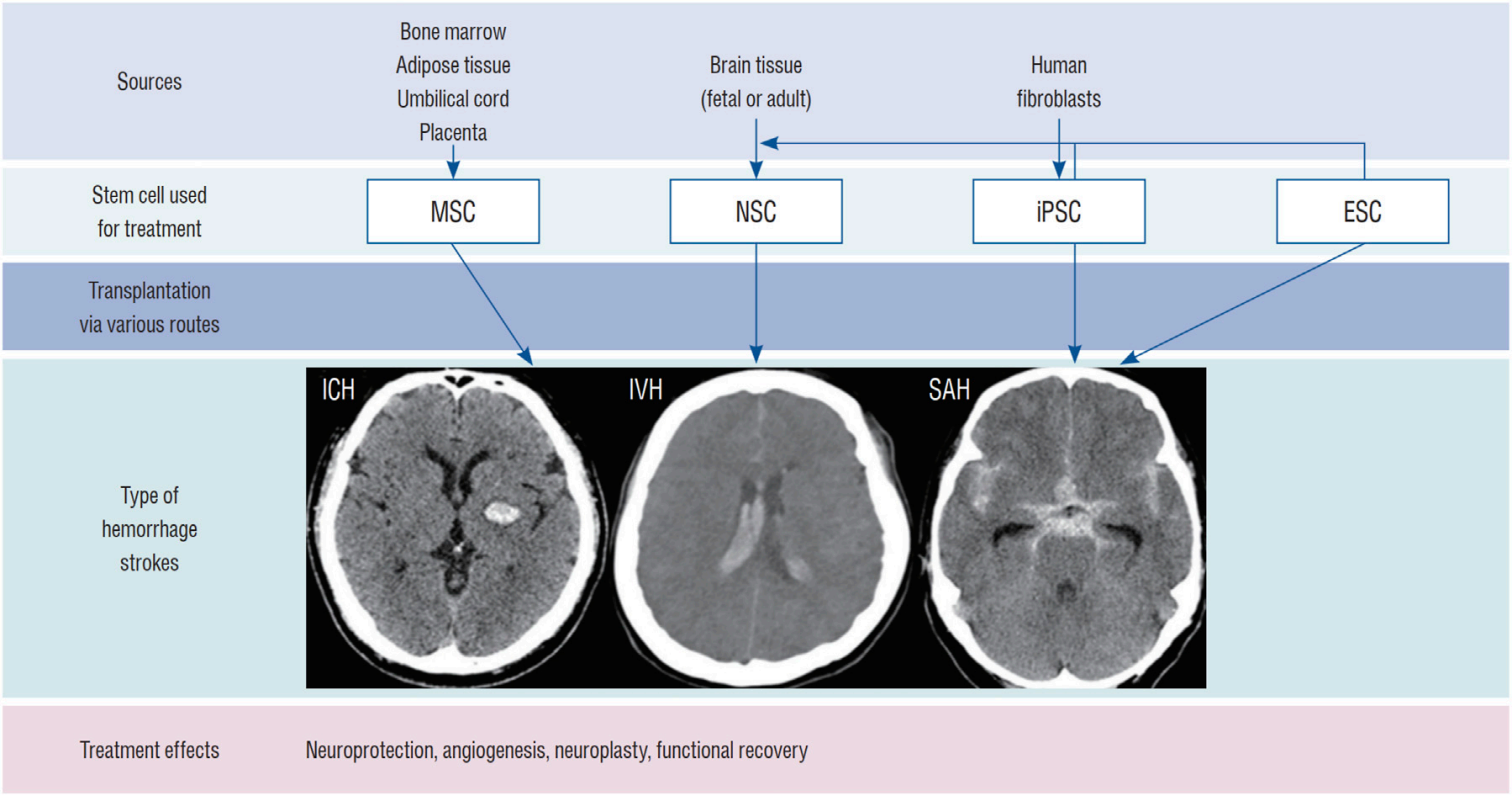
Stem cell Types for ICH Treatment: This diagram displays stem cell types for treating ICH (parenchymal hemorrhage, intraventricular hemorrhage [IVH], and subarachnoid hemorrhage [SAH]), including NSCs, mesenchymal stem cells (MSCs), induced pluripotent stem cells (iPSCs), and embryonic stem cells (ESCs).

ESCs and iPSCs can differentiate into specific cell types, but ESCs face ethical and immunogenicity challenges due to their embryonic origin (Kimbrel and Lanza, 2015), while iPSCs, developed to bypass these issues, require safety validation due to genetic modifications via viral vectors like lentiviruses and adenoviruses (Ratcliffe et al., 2013). NSCs excel in treating neurological diseases, differentiating into functional cells and releasing nerve growth factors to stimulate endogenous repair, though their deep brain location complicates harvesting. MSCs, widely studied for their ease of isolation from adult tissues, support regeneration through anti-inflammatory, angiogenic, and growth factor secretion (Ji et al., 2021). Advancing SC therapy for ICH requires integrating biomaterials, bioengineering, gene overexpression, and pretreatment strategies to boost endogenous regeneration (Kim et al., 2022). Despite promising efficacy, large-scale ICH trials and robust safety data are lacking (Kim et al., 2022) (Figure 3). Future research should optimize delivery methods, dosages, timing, and SC mass production feasibility.

## 10 The promise and challenge of NSCs treatment

### 10.1 The application prospects of neural stem cells

Spontaneous intracerebral hemorrhage (SICH) poses a major threat to human health, with current medical and surgical treatments debated for their ability to improve long-term outcomes due to extensive neuronal damage that remains difficult to repair with existing technology. Preclinical studies, including animal and cell-based research, demonstrate that NSC transplantation offers promising therapeutic benefits for ICH. NSCs replace damaged neurons, reduce inflammation, and inhibit apoptosis, positioning this emerging therapy as a potentially effective solution for post ICH recovery (Zhou et al., 2022).

Endogenous NSCs provide a safe repair option, but their limited numbers and inefficient migration and differentiation necessitate future research into effective stimulants (Zhao et al., 2022). Intrinsic repair alone struggles to regenerate lost neurons, making exogenous NSC transplantation the dominant approach. Combining epidermal growth factor (EGF) and basic fibroblast growth factor (bFGF) in serum-free cultures rapidly produces abundant NSCs, ensuring a reliable supply (Schmidt et al., 2021). Studies show that pairing hematoma clearance with NSC transplantation outperforms standalone interventions, suggesting new treatment pathways (Zhang et al., 2015). While preclinical success drives expanding clinical trials—mostly for ischemic stroke—further trials are needed to confirm NSC efficacy in ICH (Kalladka et al., 2016).

### 10.2 The application challenges

NSC neuroprotective mechanisms, likely involving inflammation modulation and apoptosis suppression, remain incompletely understood and require deeper study. Current animal and *in vitro* models, often using healthy subjects, fail to fully replicate clinical ICH pathophysiology, highlighting the need for improved models. Challenges such as transplantation timing, methods, differentiation cues, immune responses, and ethical issues persist. Despite these uncertainties, NSC transplantation represents the most promising strategy for addressing ICH-related challenges.

### 10.3 Immunological and safety challenges

A critical barrier to NSC translation is immunological rejection and safety risks. While autologous NSCs minimize rejection, allogeneic transplants can trigger T-cell mediated immune responses, as reported in several studies (Pluchino et al., 2005). Tumorigenicity is a major concern, particularly with iPSC- or ESC-derived NSCs, due to potential teratoma formation from undifferentiated cells (Yang et al., 2017; Mollinari and Merlo, 2021). Mitigation strategies include CRISPR-based gene editing for immune evasion, pretreatment with immunosuppressants, and rigorous purity checks. Ethical issues (e.g., embryonic sourcing) and manufacturing scalability (e.g., GMP-compliant production) further complicate clinical adoption, requiring regulatory oversight from bodies like the FDA.

## 11 Conclusion and future perspectives

Post-ICH interventions, whether surgical or pharmacological, have reduced mortality, disability, and improved quality of life to some degree, yet persistent neurological deficits remain difficult to fully resolve. NSC transplantation is being explored as a potential solution, though it remains largely in the preclinical animal stage, with clinical use still distant. The complex mechanisms of neuroprotection, neurogenesis, and repair following ICH drive ongoing research, with preventing neuronal death via ferroptosis and necroptosis emerging as a key focus. NSCs’ strong immunotolerance and versatile differentiation capacity make them a promising therapy for hemorrhagic stroke. However, current NSC subtypes, mostly rat-derived, differ from human cells, and debates over transplantation timing, dosage, and mechanisms of induction, differentiation, and migration require further study to optimize strategies.

While NSC transplantation appears safe and feasible in many studies, challenges persist, including ethical concerns, efficacy, complications, immune rejection, and the critical risk of tumorigenesis. Long-term safety remains contentious, with uncertainties in controlling proliferation and differentiation. Clinical research lags behind preclinical promise, with trials limited to phases I and II and no large-scale phase III studies. Extensive future trials are essential to confirm the safety and efficacy of NSC transplantation for hemorrhagic stroke. The heterogeneity in parameters (e.g., doses of 10^5–10^8 cells, acute vs. chronic timing) contributes to inconsistent results, underscoring the need for standardized protocols to close the translational gap.
